# Short-term Efficacy of Hand-Arm Bimanual Intensive Training on Upper Arm Function in Acute Stroke Patients: A Randomized Controlled Trial

**DOI:** 10.3389/fneur.2017.00726

**Published:** 2018-01-19

**Authors:** Guilin Meng, Xiuling Meng, Yan Tan, Jia Yu, Aiping Jin, Yanxin Zhao, Xueyuan Liu

**Affiliations:** ^1^Shanghai Tenth People’s Hospital, Tongji University School of Medicine, Shanghai, China; ^2^School of Computer Science and Informatics, Indiana University Bloomington Bloomington, IN, United States; ^3^Houma People’s Hospital, Shanxi, China

**Keywords:** hand-arm bimanual intensive training, stroke, upper extremity, dysfunction, randomized controlled trial

## Abstract

**Background:**

Rehabilitation training during the acute phase of stroke (<48 h) markedly improves impaired upper-limb movement. Hand-arm bimanual intensive training (HABIT) represents an intervention that promotes improvements in upper extremity function in children with cerebral palsy. This study repurposed HABIT in acute stroke patients and assessed recovery of upper extremity function when compared with a conventional rehabilitation program (CRP).

**Methods:**

In a randomized trial, 128 patients with acute stroke were assigned to the HABIT or the CRP groups. The primary endpoint was clinical motor functional assessment that was guided by the Fugl-Meyer motor assessment (FMA) and outcomes of the action research arm test (ARAT). The secondary endpoint was an improved neurophysiological evaluation according to the motor-evoked potential amplitude (AMP), resting motion threshold (RMT), and central motor conduction time (CMCT) scores over the 2-week course of therapy. In both groups, scores were evaluated at baseline, 1 week from commencing therapy, and post-therapy.

**Results:**

After 2 weeks, the HABIT group showed improved scores as compared the CRP group for FMA (51.7 ± 6.44 vs. 43.5 ± 5.6, *P* < 0.001), ARAT (34.5 ± 6.2 vs. 33.3 ± 6.3, *P* = 0.022), and AMP (1.1 ± 0.1 vs. 1.0 ± 0.1, *P* < 0.001). However, CMCT (8.6 ± 1.0 vs. 9.1 ± 0.6, *P* = 0.054) and RMT (55.3 ± 4.2 vs. 57.5 ± 4.1, *P* = 0.088) were similar when comparing between groups.

**Conclusion:**

HABIT significantly improved motor functional and neuro-physiological outcomes in patients with acute stroke, which suggested that HABIT might represent an improved therapeutic strategy as compared CRP.

## Introduction

Stroke is a common disabling health problem globally, and presentation of this condition is widely considered as one of the major causes of acquired adult disability (1–3). The clinical sequelae of acute stroke include hemiplegia, motor weakness, free exercise reduction, aphasia, hemianopia, neglect, and general cognitive dysfunction (4, 5). Physical impairment of the affected extremities includes paresis/paralysis, loss of sensory function, presentation of muscle function abnormalities, and loss of dexterity (6).

Moreover, in approximately 50% of acute stroke survivors, chronic functional impairment of the upper limbs and hands is seen (7). This impairment severely impacts the patients’ daily life and the therapeutic effect of rehabilitation training, which can dampen the quality of life of the patient following stroke (8, 9). Thus, rehabilitation of upper-limb function is a critical issue.

Previous studies have focused on poststroke rehabilitation management (10, 11), including task-oriented training, constraint-induced movement therapy (CIMT), bilateral training, error-based feedback, robotic-assisted movements, impairment oriented training, virtual reality therapy, gaming learning-based activities, mental imagery, non-invasive electrical stimulation, progressive task-specific repetitions, and skill acquisition training that is paired with motivational enhancement (12). A meta-analysis suggested that among the aforementioned approaches, the most promising includes robot-assisted therapy and CIMT (13), rather than bilateral training; however, quite convincing and novel findings provide evidence supporting bilateral trainings as effective rehabilitation protocols in stroke patients (14, 15). CIMT is a widely applied approach with a robust theoretical foundation that is based on the principle that cortical excitability increases during training and improves sensorimotor recovery (16, 17). Nevertheless, CIMT has not been consistently applied as a standard rehabilitation practice, due at least in part, to restrictions on enrollment, reimbursement, high intensity, and compliance of both the patient and clinician (18). Therefore, an approach with a similar efficacy that lacks the observed limitations is clearly needed.

Hand-arm bimanual intensive training (HABIT) is a bimanual rehabilitation approach that addresses the impairments that are specific to the upper extremity in children presenting with unilateral cerebral palsy, which had demonstrated positive outcomes that were at least comparable to that of CIMT (19). HABIT is not only based on ordinary bilateral coupling or mirror movements, but also on asymmetrical movements of both hands, which uses the principles of motor learning (i.e., practice specificity, types of practice, and feedback) and neuroplasticity (i.e., practice-induced brain changes arising from repetition, increasing movement complexity, motivation, and reward). The HABIT approach also includes increasing complexity of the functional activities that necessitate the use of both hands and repetitions to achieve functional goals (19).

Studies on HABIT in adult populations are lacking. Nevertheless, based on pediatric studies and the similar mechanisms thought to be involved in damage to the brain seen in stroke and cerebral palsy, including brain malformations, periventricular brain lesions, and middle cerebral arterial infarctions (20), it could be hypothesized that HABIT improves outcomes in adults following acute stroke. This current study thus represents the first report of HABIT as rehabilitation training in adult patients presenting with acute stroke.

The present study aimed to assess the efficacy of HABIT on motor functional recovery of the upper extremities in patients with acute stroke as compared the conventional rehabilitation program (CRP) with the intention of exploring the potential benefits of HABIT and to provide additional possibilities for optimally rehabilitating patients suffering from stroke.

## Materials and Methods

### Patients and Recruitment

This single-blind randomized trial included the evaluation of consecutively untreated patients from the outpatient and emergency departments that were diagnosed with acute stroke and hospitalized in the Department of Neurology of Shanghai Tenth People’s Hospital from May 1, 2016, to December 1, 2016.

The inclusion criteria were (1) patients with stroke that was diagnosed clinically and/or by computed tomography (CT) or magnetic resonance imaging; (2) patients who were 45–75 years of age; (3) stable vital signs and a GCS score > 8; (4) patients who suffered only one stroke and could provide informed consent personally or by proxy; (5) within 48 h from onset, evidence of persistent hemiparesis leading to impaired upper extremity function, and upper-limb muscle strength that exceeded level 2 on the Medical Research Council muscle scale; and (6) no severe cognitive disorders with a Mini-Mental State Exam score ≥ 24. The exclusion criteria were (1) presence of negligible or minimal aphasia that impeded the clear understanding of provided instructions; (2) severe cardiopulmonary complications; (3) evidence of prior stroke on the same side leading to impaired upper extremity function or intra-cerebral hemorrhage; (4) conditions that limited the use of the upper limb prior to presentation of stroke; (5) epilepsy; (6) presence of a pacemaker; (7) an intracranial implant; (8) presence of a cranial defect; or (9) a sudden worsening condition that prevented the patient from resuming rehabilitative training.

The study was approved by the local Ethics Committee of Shanghai Tenth People’s Hospital, Tongji University, Shanghai, China. The study was registered in the Chinese Clinical Trial Registry (No. ChiCTR-INR-17010469).

### Study Design

A randomized trial design was used. Patients who met the criteria were randomly allocated to the HABIT or CRP group using a computer-generated random number table. Categorization was conducted in a blinded manner by using opaque sealed envelopes containing the computer-generated table of random numbers. To determine the trial designation, the envelopes were opened sequentially by study staff, after obtaining participant consent.

Conventional rehabilitation program and HABIT were both based on comprehensive rehabilitation including physical, occupational, and speech therapies. The difference between the two approaches was observed only in terms of the physical therapy component. In addition to occupational and speech therapy, the CRP group was treated by conventional rehabilitation training, while the HABIT group was treated by HABIT, respectively. Total treatment duration was 2 weeks. All therapies were delivered by the same physical and occupational therapist who was nationally accredited by possession of approved professional licenses. Owing to the random and single-blind study design; only the evaluator and statistician were kept uninformed of the grouping procedures.

The primary endpoint was clinical motor functional efficacy according to the Fugl-Meyer motor assessment (FMA) and the action research arm test (ARAT). The secondary endpoint was an improvement in the neurophysiological evaluation according to the scores that were recorded for motor-evoked potentials amplitude (AMP), resting motion threshold (RMT), and central motor conduction time (CMCT) over the 2-week schedule.

### Intervention

Conventional rehabilitation program employed principles of impaired extremity motor and functional learning, including specific task practice and fostering problem solving with respect to position training, hemiplegia side body placement, body sensory training, bridging activities, tuck exercise, self-participation in finger interlocking, and active and passive training of joint motion as described previously (21).

Hand-arm bimanual intensive training was modified from the version previously described by others (22) and included the following guidance: (1) training in pectoral girdle control ability: strengthening the pectoral girdle muscle and improving myodynamia and stability of the pectoral girdle upon weight-bearing and against resistance conditions; (2) haptic perception training: processing bimanual training in terms of tactile sense, perception, and discrimination, and the option of using articles of different texture, shape, and size; (3) bimanual coordination training involving both sides of the body, such as putting on and taking off different clothes, and manually dressing with buttoned clothes of different shapes; and (4) functional training of the hands including writing and painting with crossing of the center line, and the use of scissors and folding paper. For example, patients were guided to fold and unfold a sheet of paper using both hands so that there was a line in the middle of the paper; drawing a symmetrical/asymmetrical picture whereby the left hand drew the left part, and the right hand drew the right part, and by practicing wrist and finger extension of the involved hand, using the uninvolved hand as a stabilizer to assist in this task and to create a template, following which the task was concluded by cutting out the figures with scissors.

The training mentioned above included the key content of structured practice with increasing bimanual hand complexity and functional activities. In addition, training emphasized the requirement to repeat bimanual cooperation tasks. Compared to Gordon’s HABIT proposal in children with hemiplegia, we reduced the primary bimanual practice time from 6 h/day to 2 h/day by deleting the need for video games, card games, and manipulative tasks.

All interventions were provided by the same professional physical therapist with >5 years of experience. Both HABIT and CRP therapies were provided as 1-h sessions. Additionally, if the exercises were completed in less than 1 h, additional exercises were performed to complete the hour. The therapeutic session were comprised of two sessions/day, and at 5 days/week for two consecutive weeks (i.e., a total of 10 week days), for a sum total of 20 h for each approach. If the patients complained of fatigue, the training was ceased and resumed after 1 h of rest to complete the 1-h required session. If the intensity was intolerable, the exercises were adjusted accordingly. All assessments were evaluated before (i.e., at baseline), and after 1 week of therapy, and after 2 weeks of therapy. The same evaluator and statistician (both blinded to grouping) evaluated and analyzed all patients.

### Primary Outcomes

#### Clinical Motor Functional Efficiency

##### Fugl-Meyer Motor Assessment

The assessment includes functional classes of upper extremity reflex activities, flexor synergy motion, extensor synergy motion, activities that were accompanied by synergy motion, disengaging movement, normal reflex action, and carpal joint stability (23). The FMA assessment contained 33 items (divided into nine domains) regarding different movements, reflexes, and coordination. Each was graded from 0 to 2 (i.e., for a maximum score of 66) where 0 = could not perform, 1 = could partially perform, and 2 = could fully perform. The FMA is considered a reliable and valid assessment of the efficiency of recovering upper extremity function in stroke patients and mainly covers the Body Function/Body Structure domain (ICF B7, Musculoskeletal and Movement Related Functions) of the International Classification of Functioning, Disability, and Health framework (ICF) (24).

##### Action Research Arm Test

The test includes subtests of grasping, gripping, pinching, and gross anatomical movements (25). Scores for each ordinal scale range from 0 to 3 (i.e., with a maximum test score of 57). The quality of the movements per item was rated on a four-point scale that ranged from no movement at all (0) to minor movement performed (1), a major movement performed (2), and movement performed completely normally (3). The patient was asked to execute the movement tasks with the hand on the table. ARAT contains predominantly ICF activity items and is complementary to the FMA.

### Secondary Outcomes

#### Neurophysiological Improvement Evaluation

Transcranial magnetic stimulation (TMS) is a neurophysiological evaluation that includes RMT (26), motor-evoked potential amplitude (AMP) (27), and CMCT (28). The TMS evaluations were performed using a MagVenture MagPro X100 transcranial magnetic stimulator (MagVenture A/S, Farum, Denmark) and parabolic coil (MMC 140 parabolic; MagVenture A/S, Farum, Denmark). Patients were positioned comfortably in a chair, with the concave surface of the coil for cranial and spinal stimulation. Electromyographic data were recorded from the contralateral abductor digiti minimi (ADM) muscle using a Medtronic Keypoint Interactive Portable device (Medtronic, Fridley, MN, USA). The RMT, AMP, and CMCT were measured in ADM muscles. The RMT was the lowest stimulus intensity for eliciting MEPs > 50 mV during a resting period. The CMCT was the latency difference between MEPs that were induced by stimulation of the motor cortex or stimulation of the seventh spinal cervical vertebra (motor root) during muscle resting state. If the response was not obtained, the stimulation intensity was 20% above the threshold or increased gradually up to 100% of the stimulator output. For AMP, the stimulus intensity was increased gradually by 5% incremental steps from the stimulation threshold until the maximal MEP amplitude was induced.

In the present study, TMS was not used as an assisted rehabilitation strategy, but rather only as an evaluation tool. The patients were placed in the resting position without contractions. In order to ensure rest, surface electrodes were used in this study.

### Data Analysis

Sample size calculations were performed based on FMA scores as a function of mean improvement of 5± with an α-value of 0.05 and a 1 − β value of 0.80, representing 20% losses at follow-up. Therefore, 60 participants in each group were required. All statistical analyses were performed with SPSS version 22.0 statistical software (IBM, Armonk, NY, USA). Mean ± SD about the mean were calculated for all continuous variables. The Kolmogorov–Smirnov test revealed that all clinical and neurophysiological parameters had a normal distribution. Differences in baseline parameters between both groups were analyzed using the Chi-square test for categorical variables and independent-sample *t*-tests for continuous variables. We explored both changes immediately after intervention and whether these changes were subsequently retained; thus, a two-way (time × group) repeated measures ANOVA was used to evaluate differences for each measure immediately after the intervention, followed by Bonferroni-adjusted *post hoc* analyses. *P*-values with an alpha value of <0.05 were considered statistically significant.

## Results

### General Characteristics of Subjects

**Figure 1:**
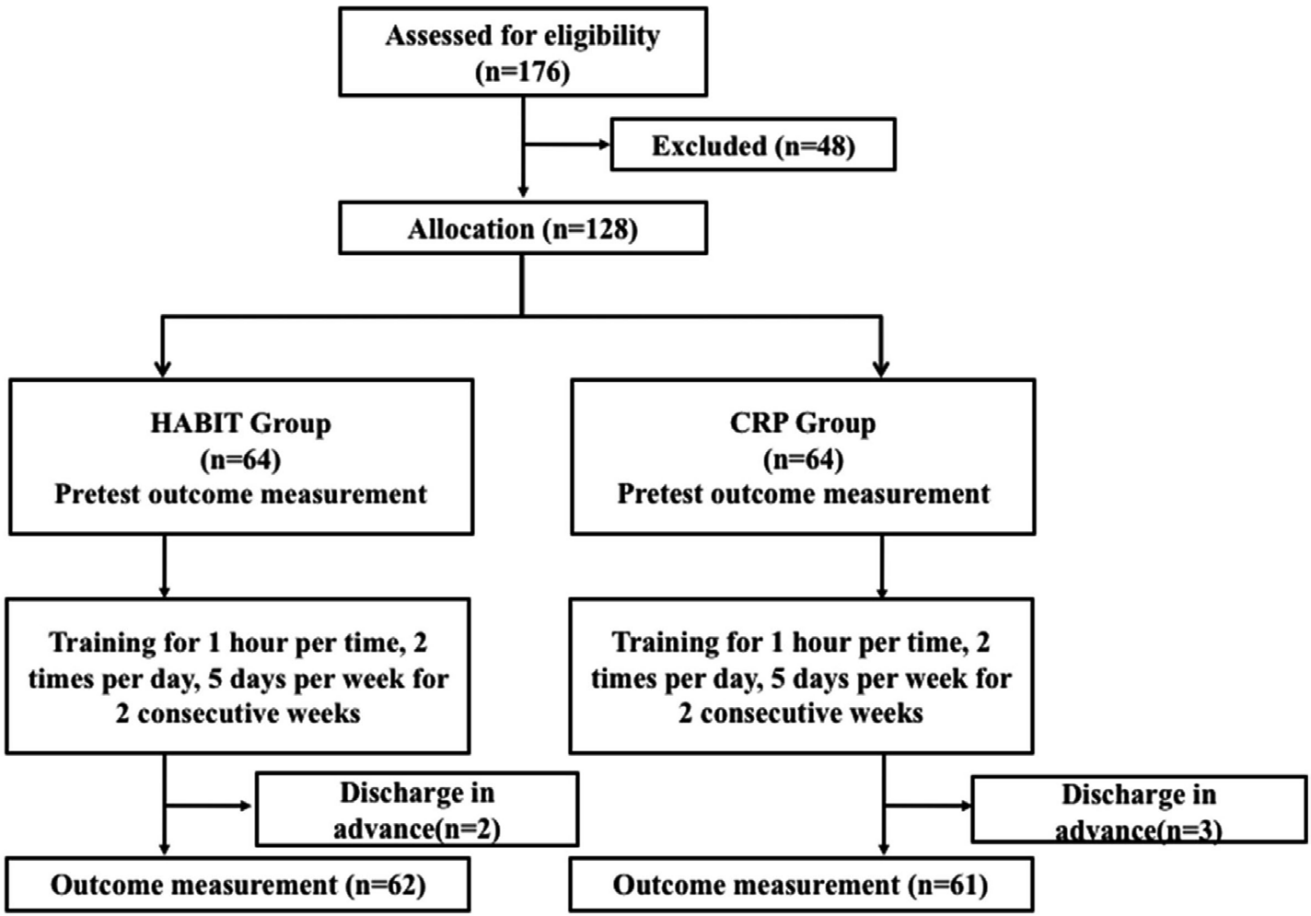
Patient flowchart.

A total of 176 patients with acute stroke were admitted to the Neurology Department from May 1, 2016 through December 1, 2016. Of these, 128 eligible study subjects were equally randomized to the HABIT and CRP group (*n* = 64/group). Two patients withdrew from the HABIT group, and three patients withdrew from the CRP group, owing to personal reasons that required advanced hospital discharge. Thus, 123 participants completed the 2-week protocol. Figure 1 presents the study flowchart. Table 1 describes the patient characteristics. The demographics of both groups were similar (all *P* > 0.05). Before treatment, no significant differences were observed between groups for FMA, ARAT, AMP, CMCT, and RMT.

**Table 1:** Demographic data of the patients.

	Hand-arm bimanual intensive therapy (*n* = 64)	Conventional rehabilitation program (*n* = 64)	*P*-value
Gender			0.596*
Male	34 (53.1%)	31 (48.4%)	
Female	30 (46.9%)	33 (51.6%)	
Age (years)	55.38 ± 6.97	55.19 ± 7.82	0.886**
Disease duration before consenting to the study (h)	8.87 ± 2.69	9.08 ± 2.35	0.609**
Side of lesion			0.327*
Right	35 (54.7%)	33 (51.6%)	
Left	29 (45.3%)	31 (48.4%)	
Lesion type			0.684*
Ischemic	50 (78.1%)	45 (70.3%)	
Hemorrhagic	14 (21.9%)	19 (29.7%)	
Fugl-Meyer motor assessment	33.25 ± 5.89	32.86 ± 5.11	0.689**
Action research arm test	30.31 ± 6.07	31.48 ± 5.94	0.272**
AMP (mV)	0.63 ± 0.12	0.63 ± 0.06	0.993**
Central motor conduction time (ms)	11.56 ± 0.85	11.53 ± 0.87	0.888**
Resting motion threshold (%)	64.35 ± 4.11	65.75 ± 4.28	0.062**

*Values are presented as mean ± SD*.

*Chi-square test.

**Student’s t-test.

*AMP was evaluated in the C3 area of the affected cerebral hemisphere*.

### Treatment Effects

The assessment scales of both HABIT and CRP groups at baseline, and at 1 week and 2 weeks of therapy are shown in Table 2. Treatment adherence was satisfactory, and all 20 targeted sessions were completed by all patients. Both groups showed statistically significant improvements of all measures (FMA, ARAT, AMP, CMCT, and RMT) from baseline to posttreatment assessment. After the first week of therapy, the HABIT group showed significant improvement in all assessment scales: FMA (*P*_time_ < 0.001, *P*_group_ < 0.001), ARAT (*P*_time_ = 0.009, *P*_group_ = 0.015), AMP (*P*_time_ < 0.001, *P*_group_ < 0.001), CMCT (*P*_time_ = 0.041), and RMT (*P*_time_ < 0.001). After 1 week, the ARAT, CMCT, and RMT results in the HABIT and CRP groups showed a plateau and did not show any additional improvement. After 2 weeks, the HABIT group had significantly improved FMA and ARAT scores (*P* < 0.001 and *P* = 0.022, respectively) than did the CRP group. A significant difference was observed in terms of AMP (*P* < 0.001) on comparing the HABIT and CRP groups after 2 weeks of therapy; however, there were no difference in terms of CMCT and RMT assessments.

**Table 2:** Comparison of assessments in both groups before and after rehabilitation.

	Group	Pre-therapy	After1 week	Post-therapy	Change of post-therapy minus pre-therapy (min, max)	*P*_(time)_ pre-therapy vs. 1 week	*P*_(time)_ 1 vs. 2 weeks	*P*_(time)_ pre-therapy vs. 2 weeks	*P*_(group)_ Hand-arm bimanual intensive training (HABIT) vs. conventional rehabilitation program (CRP) after 1 week	*P*_(group)_ HABIT vs. CRP post-therapy
Fugl-Meyer motor assessment	HABIT	33.25 ± 5.89	43.30 ± 5.95	51.73 ± 6.44	18.48 (15, 31)	<0.001	<0.001	<0.001	<0.001	<0.001
	CRP	32.86 ± 5.11	39.42 ± 4.91	43.45 ± 5.61	10.59 (8, 19)	<0.001	<0.001	<0.001		
Action research arm test	HABIT	30.31 ± 6.07	33.27 ± 6.10	34.47 ± 6.22	4.16 (3, 5)	0.009	0.054	<0.001	0.015	0.022
	CRP	31.48 ± 5.94	31.84 ± 5.91	33.34 ± 6.26	1.86 (−2, 5)	0.027	0.082	0.017		
AMP(mV)	HABIT	0.63 ± 0.12	0.99 ± 0.13	1.14 ± 0.13	0.51 (0.55, 0.70)	<0.001	<0.001	<0.001	<0.001	<0.001
	CRP	0.63 ± 0.06	0.85 ± 0.10	0.96 ± 0.05	0.33 (0.15, 0.48)	0.032	0.653	0.012		
Central motor conduction time (ms)	HABIT	11.56 ± 0.85	9.85 ± 0.87	8.64 ± 0.95	−2.92 (−3.99, −2.04)	0.041	0.203	0.017	0.061	0.054
	CRP	11.53 ± 0.87	10.19 ± 0.55	9.06 ± 0.59	−2.47 (−2.09, −0.52)	0.046	0.142	0.033		
Resting motion threshold (%)	HABIT	64.35 ± 4.11	59.36 ± 4.07	55.32 ± 4.19	−9.03 (−9.95, −8.04)	<0.001	<0.001	<0.001	0.041	0.088
	CRP	65.75 ± 4.28	61.40 ± 4.63	57.46 ± 4.09	−8.29 (−9.56, −7.98)	0.020	0.210	0.027		

*Values are presented as mean ± SD*.

### Safety

No adverse event occurred during this trial.

## Discussion

This study aimed to explore the efficiency of the repurposed HABIT strategy at improving upper arm function in adult patients with acute stroke. The results showed that HABIT and CRP improved upper arm function in acute stroke patients. Positive changes in all outcomes were seen in both the HABIT and CRP groups, with more effective results seen after 2 weeks than following 1 week of therapy. A direct comparison between baseline and post-therapy assessment scores revealed marked improvements in the HABIT group as compared to the CRP group, with positive effects seen for FMA, ARAT, and AMP emerging with the same therapeutic duration. The findings revealed that HABIT might represent a more clinically effective treatment strategy than CRP for adult patients presenting with acute stroke. The impact of HABIT on neurophysiological evaluation requires additional research. Furthermore, we propose that not only AMP, but also CMCT and RMT should be considered together.

It is speculated that motor function recovery of patients with acute stroke is a time-sequential process, and the assessments of motor, sense, balance coordination, joint function, and motion amplitude of the patient using FMA at different time-points provides an assessment of the time-dependent recovery of the patient (29). FMA has great reliability and validity and is widely applied in the assessment of arm function in patients with acute stroke (8). FMA is usually used in combination with ARAT, which evaluates the ability to handle small items and gross motor skills of the upper extremities (30). ARAT is regarded as a specific assessment for evaluating upper extremity dysfunction. AMP can effectively predict the prognosis of patients with acute stroke (31). After onset of acute stroke, a higher amplitude in AMP is a reliable indicator of improved motor recovery and functional outcomes (32). Moreover, motor recovery is closely related to an improvement in corticospinal tract conduction, and a shortened CMCT indicates predominant motor recovery (33). Therefore, in this study, FMA, ARAT, AMP, RMT, and CMCT were used to assess therapeutic effectiveness following HABIT and CRP with the aim of identifying a superior approach.

There is a close relationship between neurophysiological and clinical evaluation after stroke in terms of motor recovery of the upper extremity, while the results concerning the AMP, CMCT, and RMT sensitivity and specificity were inconsistent, most likely due in part to differential degrees of paresis and follow-up periods (34). For instance, patients with severe paresis (MSC muscle score 0–1) of the upper extremity, who experience motor recovery, are likely to have an early AMP improvement, even when clinical examination cannot be detected for motor recovery (35). Besides, some studies show that TMS parameters are more sensitive in longer follow-up periods in terms of motor function recovery in patients with acute ischemic stroke (36). In this study, there seemed to be a clear association between AMP and motor recovery than CMCT and RMT in a two-week rehabilitation therapy—an observation that indicated the superior predictive ability of AMP for functional recovery over a short time period.

Since bimanual intensive training has scarcely been systematically investigated in acute stroke, the eventual benefits in stroke rehabilitation remain poorly understood. The present study is the first preliminary exploration of a possible repurposing of the HABIT approach in adults with acute stroke. Therefore, evidence from previous studies is lacking at this point for a meaningful comparative analysis and assessment. Nevertheless, we can still obtain a perspective based on other bilateral trainings and application of HABIT in other relevant populations (37), which have shown the capacity for intensive training to improve hand function after HABIT therapy, which is consistent with motor learning theories. Another bilateral arm training system including kinematic behavior, sub-movement and bimanual coordination, showed acceptable feasibility and sensitivity in terms of manipulation function of the paretic arm and coordination of the bilateral upper limbs (38). Besides, bilateral priming with active-passive movements promotes rebalancing of corticomotor excitability and would be expected to accelerate upper-limb recovery at the subacute stage as measured by ARAT (39).

In another single-blinded randomized controlled trial, unilateral and bilateral upper-limb trainings were compared. In this study, the bilateral group showed greater movement harmony and larger movement amplitudes, which clearly benefited from the influence of interlimb coupling (15). Furthermore, the bilateral therapies of mirror movements (40), which focused on the coupling with the impaired extremity (although not placing the bimanual coordination at the first priority), still showed acceptable rehabilitation efficacy.

The human corticospinal system undergoes reconstruction after stroke, manifesting as functional recovery, which leads to the hypothesis that HABIT could improve upper extremity function after acute stroke. Additional studies aimed at comparing multiple approaches and focusing on mechanisms of bimanual intensive motor recovery should be performed.

In the present study, the intervention lasted 2 weeks and displayed a less than 5% drop-out rate. This situation is probably explained by hospitalized patients being inherently more compliant than outpatients. Moreover, a rest period was allowed when the patients were unable to complete a session, which probably helped patient retention in the program. Furthermore, all patients included in the present study had a relatively good prognosis as the muscle strength was above level two, which indicates a relatively stable condition of the patients, which did not become aggravated or decline after admission.

As a single center randomized controlled clinical trial, this study was mainly a preliminary exploration to ensure safety before designing and implementing a larger scale clinical trial. Although the targeted population in this study was patients with acute stroke, the results suggested that a longer follow-up might be necessary to record the neurophysiological outcomes in the subacute and chronic phases. In addition, comparison between HABIT and the well-known CIMT are certainly planned for at our institute when additional studies begin in the near future. The long-term prognosis of patients after HABIT and whether HABIT is suitable for all kinds of upper extremity dysfunction necessitates further investigation.

However, there are still some limitations to this study. The retention tests should be considered to test whether patients have learned the task at varying time periods to determine at what extent particular motor skills have been retained such as during the weeks following training. Another important consideration is that the same therapist may lead to a controlling bias, but it could also be a strength since it ensures the uniformity of treatment among patients. Besides, depression should be assessed as one potential confounder in the baseline data since depression affects the motivation of the patient to fully participate in and benefit from rehabilitation; further, depression represents a notable risk factor for negative rehabilitation efficacy (41). Finally, any measures from the participation end of the ICF scale were lacking in the current study.

## Conclusion

Compared with CRP, the HABIT approach led to an improved motor performance and some neurophysiological improvements of the upper limb in adult patients who presented with acute stroke. These results suggest that HABIT might be a more effective treatment strategy in stroke than CRP.

## Author Contributions

GM, YZ, and XL conceived and designed the study. GM wrote the manuscript. XM, JY, and YT performed the research and collected data. AJ analyzed the data collected from the primary database. All authors read and approved the final manuscript.

## Conflict of Interest Statement

All authors declare that they have no conflict of interest with regard the contents of this article or in the conduct of the study.

## Abbreviations

HABIT, hand-arm bimanual intensive training; CRP, conventional rehabilitation program; MRC, Medical Research Council; ICF, International Classification of Functioning, Disability and Health framework; CT, computed tomography; MRI, magnetic resonance imaging; MMSE, mini-mental state exam; FMA, Fugl-Meyer motor assessment; ARAT, action research arm test; AMP, motor-evoked potentials amplitude; RMT, resting motion threshold; CMCT, central motor conduction time; MSB, movement science-based treatment; IOT, impairment oriented training; CIMT, constraint-induced movement therapy; ADM, abductor digiti minimi.
